# Efficacy of LMS for teaching biochemistry to medical students in India during the CoVid-19 pandemic

**DOI:** 10.6026/97320630019840

**Published:** 2023-08-31

**Authors:** Shashidhar KN, Harish Rangareddy, Munilakshmi U

**Affiliations:** Department of Biochemistry, Sri Devaraj Urs Medical College, Constituent College of Sri Devaraj Urs Academy of Higher Education and Research, Kolar – 563103, Karnataka, India

**Keywords:** Learning curve, undergraduate medical education, COVID-19

## Abstract

During the pandemic, medical education shifted to online platforms, using the Learning Management System (LMS) for lectures, video recordings, e-resources, and assessments. An inductive qualitative study was conducted among I MBBS students for Biochemistry to assess LMS acceptance and performance. Out of 150 students, 99 responded with 70.7% finding LMS effective, 67.74% agreeing it was user-friendly, and 52.8% satisfied with the grading system. Challenges included internet connectivity, but overall, students' feedback indicated LMS acceptance with academic flexibility, highlighting its potential to enhance medical education, especially during times when e-learning becomes essential.

## Background:

Howlett *et al.* defined "Electronic (e) or online learning as use of electronic technology and media to deliver, support and enhance both learning and teaching and involves communication between learners and teachers utilizing online content" [1]. Indian Medical Graduate (IMG) of the 21st century needs to update with latest technologies to ensure flexibility particularly during the pandemics and unexpected emergencies. There is a greater need for educators, students and clinicians to update their skills, and remain abreast of the changing healthcare environment and remain "digitally literate" [2]. Digital literacy is the capacity to use digital technology, communication tools or networks to locate, evaluate, use and create information and the ability to understand and use information in multiple formats from a wide range of sources when it is presented via computers [3]. COVID-19 pandemic has had a profound impact on medical education worldwide, necessitating the adoption of alternative teaching methods to ensure the continuity of learning due to the suspension of in-person classes and clinical rotations. This disruption affected the hands-on learning experiences and direct interactions between students and faculty, which are integral to medical education [4]. To mitigate the impact of the pandemic, educational institutions rapidly transitioned to remote learning modalities. Traditional lectures, seminars, and small-group discussions were replaced with online platforms, including Learning Management Systems (LMS), video conferencing, and other virtual collaboration tools [5]. Learning Management System (LMS) is a software application, provides the framework for Teaching, Learning and Evaluation (TLE) [5]. The transition to remote learning has highlighted the digital divide among students, with disparities in access to reliable internet connections, devices and conducive-learning environments [6].The exploration and implementation of innovative teaching methods have become imperative to ensure the continuity of medical education and prepare future healthcare professionals to navigate similar crises [7]. Assessing the effectiveness of the LMS in teaching Biochemistry amid the pandemic is crucial for ensuring quality education, evaluating concept delivery, and identifying effective teaching strategies in the virtual environment. It impacts student engagement, motivation, and participation, guiding institutional decision-making and future technology integration in medical education [6]. Therefore, it is of interest to evaluate the effectiveness of a Learning Management System (LMS) in delivering Biochemistry education to first-year MBBS students during the COVID-19 pandemic. Specifically, it is of interest to assess the impact of the LMS on student learning outcomes, engagement, and satisfaction, as well as explore any challenges or limitations associated with its implementation.

##  Materials and Methods:

This is an inductive Qualitative study based conventional feedback analysis conducted in the 2021-22 academic year. Universal sampling was employed, encompassing all first-year medical students during the Academic year 2021-22, to ensure comprehensive inclusion in the study. The orientation to the Learning Management System (LMS) took place during the Digital Literacy sessions of the foundation course, providing the students with necessary guidance for its usage. The SOP was shared with the students who were able to access the LMS remotely. Feedback on the institutional learning management system was obtained using Google forms after the internal assessments using a structured questionnaire containing questions using a 5-point Likert scale (strongly disagree to strongly agree)and analysis was based on qualitative metrics. Respondents included first year MBBS students n= 99/150 (66%) participants, n=51/150 (44%) did not participate probably due to the perceived stress of the summative examination that followed.

## Standard Operating Procedure of the LMS:

[1] Type the Academy URL in the web browser

[2] Confirm the Academy website

[3] In the left quick launch icons click the LMS icon and confirm the login to LMS

[4] Enter the LMS with login credentials by following the self-explanatory instructions

[5] Faculty to upload and edit online content

[6] Students to access online content

## Results:

**Figure 1:**
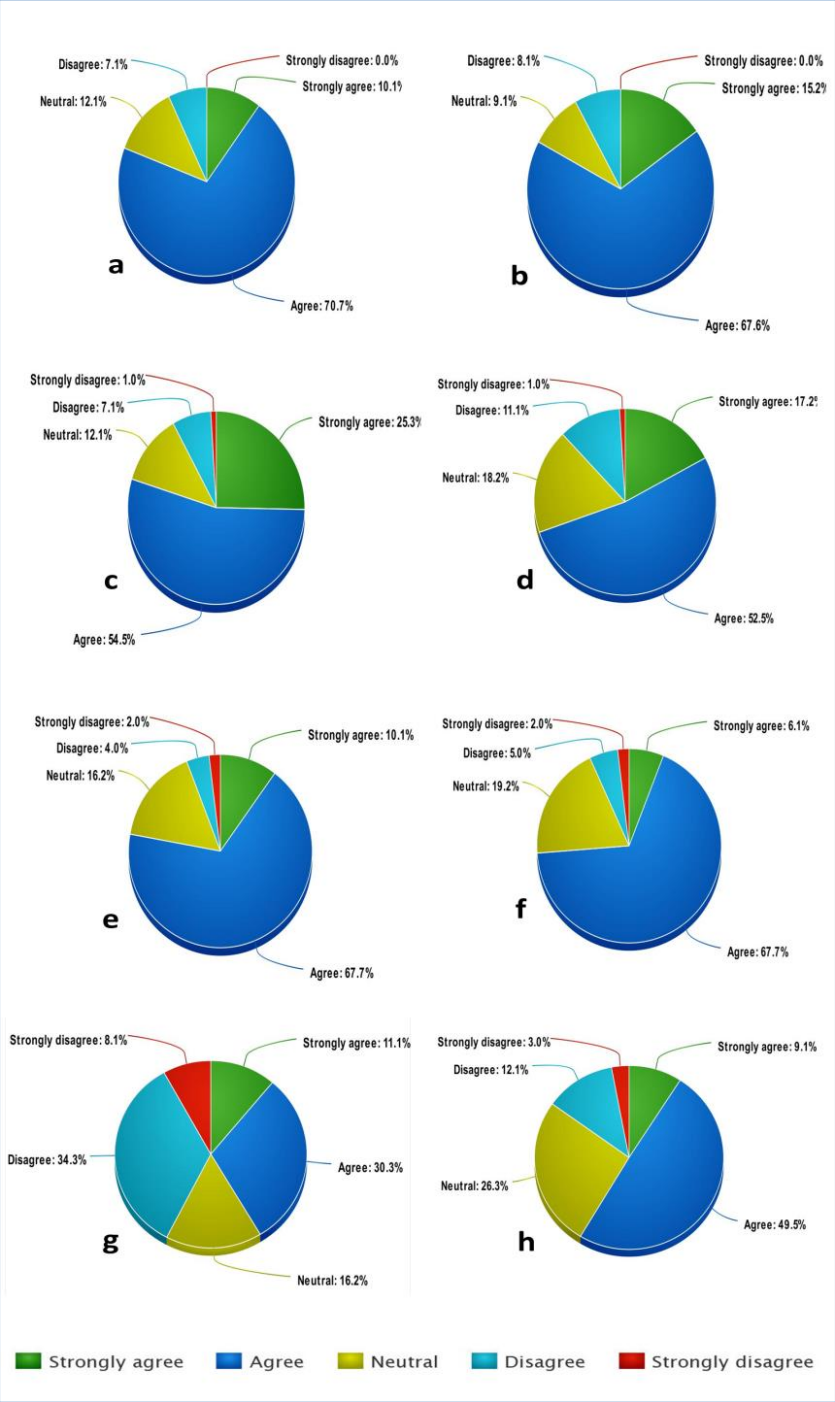
Feedback analysis (a) Overall LMS is Effective (b) LMS is User Friendly (c) Completion of Study Tasks on LMS is Time bound (d) Grading System on LMS is Satisfactory (e) Course content on LMS is clear and Comprehensible (f) Navigating content on LMS is Self- explanatory (g) Virtual Teaching is Better than Live Teaching (h) Virtual Teaching is Adjunct to Live Teaching

The demographic included boys n=55, girls n=44. The average use of LMS was 5 to 9 hours. The theme of overall acceptance of the LMS emerged from the qualitative feedback provided by first-year MBBS students. It represents their general satisfaction and positive perception of the LMS as a tool for delivering Biochemistry education during the COVID-19 pandemic as evident from the feedback analysis depicted in Figure 1.

Identified themes from the conventional feedback analysis

"The availability of Class PowerPoint presentation whenever I need it"

"We can read whenever we want to"

"Can assess topics at any time"

"Poor internet connectivity"

Students' feedback indicated overall LMS acceptance, highlighting its potential for academic flexibility in medical education and enhancing the learning experience, especially during essential e-learning periods, despite challenges related to internet connectivity issues.

## Discussion:

The study findings offer crucial insights into LMS usage in medical education, with implications for future implementation and recommendations to enhance effectiveness as follows:

Findings indicate that 67.74% of participants comprising of 55.6% female and 44.4% male first year MBBS students found the LMS user-friendly but suggest room for improvement. To enhance user-friendliness, clear instructions, streamlined navigation, and user training are recommended. Setting time-bound tasks was perceived positively by 54.5% of participants, but flexibility should be considered. Recommendations include reasonable timeframes and allowing flexibility when possible. 52.8% of participants were satisfied with the grading system, but ensuring consistency and transparency is essential through clear criteria and timely feedback. While the LMS enables theoretical knowledge and demonstration, challenges in performing competently require incorporating practical learning like virtual simulations and interactive scenarios. Integrating the LMS with live teaching is crucial (49.5% positive), utilizing pre-recorded lectures, online discussions, and collaborative activities. Internet connectivity issues and skill acquisition challenges call for offline resources, hands-on sessions, and addressing infrastructure limitations. Our study's demographics mirrored those of Dash *et al*, with a majority of female participants (61%) and a significant representation of males (34.1%) [6]. Dash *et al*. observed that 80.5% of students found accessing class notes through Google Classroom to be easy, indicating the platform's accessibility for educational resources. This result was consistent with students' perception of the helpfulness of YouTube videos posted on the platform, with an equal percentage (80.5%) expressing satisfaction. However, a noteworthy proportion (17.1%) remained neutral in their assessment [6]. One intriguing finding by Dash *et al*. was the use of Google classroom as a LMS, with a curriculum designed to incorporate elements of blended learning through Google Classroom with strongly positive outcomes which is probably attributable to the face-to-face interactions Dash *et al* had over an extended period of the academic block with the students [6]. Gaur U *et al*. in their narrative review examined the challenges and opportunities faced by medical schools in implementing remote learning for basic science teaching in response to the COVID-19 crisis. It discussed the disruptions caused by the pandemic on face-to-face teaching, the incorporation of online learning in the curriculum, and the potential implications of a lack of hands-on training during the preclinical years on future clinical performance [8]. Data also highlight the impact of the COVID-19 pandemic on medical education, specifically concerning the transition to remote learning and the challenges and opportunities it presents. The overall acceptance of the LMS among first-year MBBS students reflects several important factors contributing to its effectiveness in teaching Biochemistry during the pandemic.

## Students' perspective:

Students appreciated the flexibility and convenience offered by the LMS, allowing them to access learning materials at their preferred pace and time, accommodating various learning styles and individual schedules. The centralized platform provided comprehensive access to course materials, including lecture slides, multimedia resources, and supplementary readings, enhancing students' engagement and understanding of Biochemistry concepts. Despite physical distance, the LMS facilitated interactive learning experiences through features like discussion forums, chat functions, and virtual quizzes, promoting student engagement, collaboration, and interactions with peers and faculty members. Moreover, the LMS contributed to a supportive learning environment, enabling students to feel connected and supported, even without face-to-face interactions, as online communication tools allowed them to seek clarification and receive timely feedback from instructors.

## Faculty perspective:

Online education presents faculty members with the need to enhance competency in pedagogy, technology, and content knowledge. Challenges in pedagogy include adapting teaching strategies to engage students effectively and addressing diverse learning needs. Faculty members may also encounter challenges related to technology proficiency and troubleshooting technical issues. Adapting content for online delivery and staying current with evolving research are crucial challenges in the area of content knowledge. Additional major challenges include limited technological skills, poor time management, and lack of infrastructure. Overcoming these challenges requires professional development, institutional support, collaboration, and a commitment to continuous improvement, including training programs and technical assistance to create a supportive online learning environment [9].

## Advantages of LMS:

LMS platforms offer numerous advantages, including user-friendliness, seamless navigation, and easy access to course materials. The centralized repository of resources, such as lecture notes, multimedia presentations, and interactive quizzes, enhances self-directed learning and a deeper understanding of Biochemistry concepts. Moreover, interactive features like discussion forums and collaborative tools foster engagement and interaction, enabling students to ask questions, participate in discussions, and collaborate with peers [10]. The flexibility in learning allows students to access materials and complete assignments at their convenience, accommodating various learning preferences and promoting inclusivity in the learning environment [11].

## Disadvantages of LMS:

The effectiveness of the LMS in teaching Biochemistry depends on reliable internet access and appropriate devices, but technical challenges like connectivity issues and hardware limitations may hinder consistent engagement with course materials. The absence of in-person interaction may affect aspects of student learning, such as building rapport with instructors and classmates or participating in hands-on laboratory activities. Instructors managing large student cohorts through the LMS may face challenges in providing personalized support and timely feedback, leading to potential reductions in instructor availability [9]. Additionally, some students may experience a learning curve in adapting to the LMS functionalities, and the digital divide may exacerbate inequalities in engagement due to disparities in technology access. The limited non-verbal communication within the LMS may impact nuanced understanding of complex Biochemistry concepts that often rely on non-verbal cues for comprehension.

## Conclusion:

While the overall acceptance of the LMS was evident among the first-year MBBS students, it is important to acknowledge that individual experiences and perspectives may vary. Some challenges or areas for improvement might have been identified by students within this theme. It is crucial to consider these nuances and address any limitations or concerns to further enhance the effectiveness of the LMS in teaching Biochemistry.
